# Flexible intubating video endoscope-guided determination of optimal oral endotracheal tube depth in infants: a prospective observational study

**DOI:** 10.3389/fmed.2026.1749293

**Published:** 2026-03-13

**Authors:** Kun Yue, Xiaochun Peng, Jingru Wang, Yuanling Xu, Yingying Sun, Yin Xia

**Affiliations:** 1 Department of Anesthesiology and Perioperative Medicine, Anhui Provincial Children’s Hospital, Hefei, Anhui, China; 2 Department of Anesthesiology and Perioperative Medicine, The Children’s Medical Center of Anhui Medical University, Hefei, Anhui, China

**Keywords:** endotracheal tube, flexible intubating video endoscope, formula method, infants, intubation depth, neonates

## Abstract

**Objectives:**

To assess the accuracy of Advanced Pediatric Life Support (APLS) and Neonatal Resuscitation Program (NRP) formulas for predicting oral endotracheal tube (ETT) depth in Chinese infants undergoing elective surgery, and to develop a flexible intubating video endoscope (FIVE)-verified predictive formula for this population.

**Methods:**

In this prospective study, 189 infants (including term neonates and infants aged 1–12 months) who required oral intubation for elective surgery were enrolled. Demographics were recorded, and ETT depths were calculated using APLS and NRP formulas. A reference insertion depth was determined using FIVE, with the tube tip positioned 1 cm above the carina (a pragmatic reference position rather than a universal “ideal”). Correlations between patient characteristics and optimal depth were assessed, and new formulas were developed by linear regression.

**Results:**

In neonates, FIVE-confirmed depth correlated with height (*r* = 0.670, *P* < 0.001), weight (*r* = 0.488, *P* < 0.001), and body surface area (BSA) (*r* = 0.536, *P* < 0.001). In infants aged 1–12 months, stronger correlations were found with height (*r* = 0.952, *P* < 0.001), weight (*r* = 0.895, *P* < 0.001), BSA (*r* = 0.926, *P* < 0.001), and age in months (*r* = 0.871, *P* < 0.001). APLS and NRP formulas produced deeper predicted depths than the FIVE-referenced depth in 12.5% of neonates and 30.1% of older infants. New predictive formulas were: infants 1–12 months: depth (cm) = 4.5 + 0.1 × height (cm).

**Conclusion:**

The weight-based APLS formula may be less applicable to Chinese infants undergoing elective surgery. A height-based formula demonstrated closer agreement with the FIVE-referenced depth. Because the model was developed and assessed in the same cohort, it should be considered preliminary and requires independent external validation (preferably multicenter) before widespread clinical use, particularly in non-elective or critically ill populations.

## Highlights

1. Flexible Intubating Video Endoscope (FIVE)-confirmed optimal depth showed strong correlation with infant height.
2. New height-based formulas improved the accuracy of intubation depth prediction in infants.
3. Height-based estimation provides a simple bedside option to support depth selection, pending external validation and outcome evaluation.

## 1 Introduction

Endotracheal intubation is the primary method for establishing an artificial airway and enabling mechanical ventilation in infants undergoing general anesthesia (1). Accurate determination of endotracheal tube (ETT) depth is critical to prevent bronchial intubation, carinal stimulation, and accidental extubation (2). In children, tracheal length-from the glottis to the carina-changes with age and varies considerably among individuals of the same age group (3). Unlike adults, the margin for error in ETT depth in infants is minimal (4), making precise placement essential during perioperative airway management.

Current clinical practice commonly applies formulas recommended in the Advanced Pediatric Life Support (APLS) course (5, 6): for children older than 1 year, the aged-based formula: age (years)/2 + 12; for children aged 1 year or younger, the weighted-based formula: weight (kg)/2 + 8. In accordance with the 7th edition of the Neonatal Resuscitation Program (NRP) guidelines (7), we used the formula of body weight (kg) plus 6 cm to preliminarily estimate the endotracheal tube depth in neonates (8). However, when applying these formulas, the incidence of excessively deep or shallow ETT placement can be as high as 68% (9). For infants under 1 year of age, prediction accuracy remains limited, even with newer approaches based on body surface area (BSA), nasoseptal-ear length, and other anthropometric parameters (8, 10). Even when combined with bilateral lung auscultation to determine ETT depth (6), the possibility of excessively deep intubation cannot be ruled out (11). Imaging methods can accurately determine tube tip location but are impractical for routine anesthesia due to time and resource constraints (1). Although ultrasound, utilizing transtracheal, diaphragmatic, and pleural approaches, can assess the depth of ETT placement (12), it fails to determine the exact position of the tube within the trachea (13).

A flexible fiber-optic bronchoscope (FOB), referred to here as a flexible intubating video endoscope (FIVE), allows direct visualization of airway anatomy and localization of the ETT tip relative to the carina (12, 14). Despite these advantages, reports on FIVE-guided depth determination in infants remain scarce, likely due to limitations in equipment availability, operational expertise, and sterilization protocols. Therefore, this study aimed to compare APLS- and NRP-predicted oral ETT depths with a FIVE-referenced depth (tube tip positioned 1 cm above the carina) in term neonates and infants aged 1–12 months undergoing elective surgery, and to derive a calculation formula suitable for this Chinese cohort based on FIVE-referenced clinical data.

## 2 Materials and methods

### 2.1 Ethical approval

This study was approved by the Medical Ethics Committee of Anhui Provincial Children’s Hospital, China, on November 01, 2023 (approval number: EYLL-2023-036) and was registered at http://www.chictr.org.cn/ (trial number: ChiCTR2300078502; December 11, 2023). Written informed consent was voluntarily obtained from the parents or legal guardians of all participating infants. This single-center prospective observational study was conducted at Anhui Provincial Children’s Hospital, China, from December 2023 to December 2024.

### 2.2 Participants

This study enrolled 189 infants (including term neonates and infants aged 1–12 months) who underwent oral tracheal intubation in the operating room under general anesthesia between December 2023 and December 2024 in the Department of Anesthesiology and Perioperative Medicine at Anhui Provincial Children’s Hospital. Exclusion criteria were as follows: (1) known or suspected difficult airway, or presence of laryngeal or tracheal lesions; (2) restricted head and neck mobility; (3) severe cardiopulmonary disease, including asthma or congenital heart disease; (4) preterm birth (<37 weeks’ gestation) and/or low birth weight (<2500 g); (5) prior tracheal intubation before admission to the operating room.

### 2.3 Anesthesia and data measurement

**FIGURE 1:**
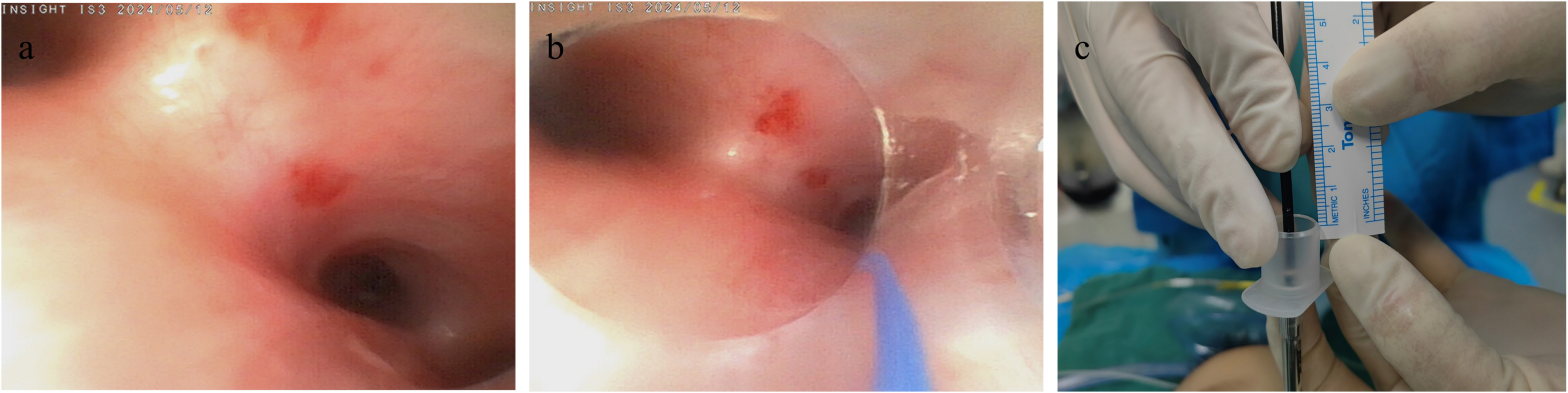
Intubation measurement procedure guided by FIVE. **(a)** Carina; **(b)** 1 cm above the carina; **(c)** measurement of the 1 cm distance.

Upon entering the operating room, each child was placed in the supine position with a shoulder roll positioned under the shoulders to achieve the sniffing position. Standard monitoring was applied, including electrocardiography, non-invasive blood pressure, pulse oximetry, and end-tidal carbon dioxide. Anesthesia induction was achieved with propofol (2–3 mg/kg), sufentanil (0.2 μg/kg) or remifentanil (1 μg/kg), and cisatracurium (0.15 mg/kg) or mivacurium (1 mg/kg). The size of the uncuffed endotracheal tube was selected primarily according to the child’s age (in months) (12). The internal diameters (IDs) of the ETTs used were 3.0, 3.5, 4.0, 4.5, and 5.0 mm. Final tube size was confirmed clinically, and the tube was changed if necessary. For infants under 1 year of age, the intubation depth was initially calculated using the APLS formula (5, 6): weight (kg)/2 + 8 cm. For neonates, the depth was calculated according to the NRP formula (7): weight (kg) + 6 cm. After achieving adequate muscle relaxation, oral tracheal intubation was performed using a standard laryngoscope. The insertion depth was first set according to the value obtained from the guideline formula. Bilateral lung auscultation was then performed, and the tube position was adjusted if breath sounds were asymmetrical. The measurement at the level parallel to the upper lip (or incisors) was recorded, rounded down to the nearest 0.5 cm, and documented as the formula-based intubation depth. Subsequently, a FIVE (Insight iS3, 2.2 mm, Guangdong, China) was used to visualize the carina, and the ETT tip was positioned 1 cm above the carina (9). For the purpose of standardizing FIVE-guided depth determination, the prespecified target position was an ETT tip 1 cm above the carina (used as the reference position for analyses). The corresponding measurement at the level parallel to the upper lip was recorded as the FIVE-guided intubation depth (Figure 1). If the distance from the tube tip to the carina measured via the FIVE through the endotracheal tube is less than 1 cm or the tube tip has passed beyond the carina, it indicates that the intubation is deeper than the prespecified target. The markings on the ETT are spaced 1 cm apart; if the intubation depth is not a multiple of 0.5 cm, it is rounded down to the nearest 0.5 cm increment as the final intubation depth. The child’s month of age, gender, weight, height, and BSA were collected and recorded. Body length (height) was measured in the supine position using a calibrated infantometer by two trained staff members (one stabilizing the head in a neutral position and the other extending the legs and aligning the heels). Two measurements were obtained and averaged; if the difference exceeded 0.5 cm, a third measurement was performed and the mean of the two closest values was recorded (15). Height was documented to the nearest 0.1 cm. The BSA was estimated by the commonly used Mosteller formula with the aid of body weight and height: BSA (m^2^) = (weight (kg) height(cm)/3600) (10).

### 2.4 Statistical analysis

This study employed a paired design. The primary outcome was to evaluate the agreement between ETT depth predicted by established formulas and the ETT depth determined by the FIVE. The ETT depth obtained via FIVE was considered the reference standard for this comparison. This study was designed to assess measurement agreement and to derive a prediction model; clinically meaningful outcomes (e.g., hypoxemia, mainstem bronchial intubation, need for ETT repositioning, atelectasis, pneumothorax) were not prespecified endpoints and were not collected. Based on the results of a pilot study, the estimated mean difference was 0.40 cm with a standard deviation of 1.54 cm. Assuming a two-sided significance level (α) of 0.05 and a power of 0.9, the required sample size was calculated to be 156 participants using PASS software, version 15.0 (NCSS, LLC, Kaysville, Utah, USA). To account for an anticipated 20% dropout rate, a total of 195 participants were planned for recruitment. Statistical analyses were performed using SPSS software, version 25.0 (IBM Corp., Armonk, NY, USA). Categorical variables were summarized as counts and percentages. Continuous variables with a normal distribution were expressed as mean ± standard deviation (SD), and those with a non-normal distribution were expressed as median (interquartile range, 25%–75%). A *P*-value < 0.05 was considered statistically significant. Spearman’s rank correlation analysis was used to evaluate the associations between FIVE-guided tracheal intubation depth and the child’s age (months), height, weight, and BSA. Variables showing statistically significant correlations in the Spearman analysis were then entered as independent variables into a linear regression model, with FIVE-guided intubation depth as the dependent variable, to develop a predictive formula for pediatric tracheal intubation depth.

Agreement between the FIVE-guided intubation depth and the depths estimated by the NRP and APLS formulas was assessed using the Bland-Altman method. For each patient, the mean difference (mean_diff) between the FIVE-guided intubation depth and the formula-calculated depth was calculated. The mean_diff and standard deviation (sd_diff) across patients were then summarized, adjusting for within-patient correlation.

The Bland-Altman limits of agreement (LOA) with a 95% confidence interval were defined as ±1.96 × sd_diff.

## 3 Results

**FIGURE 2:**
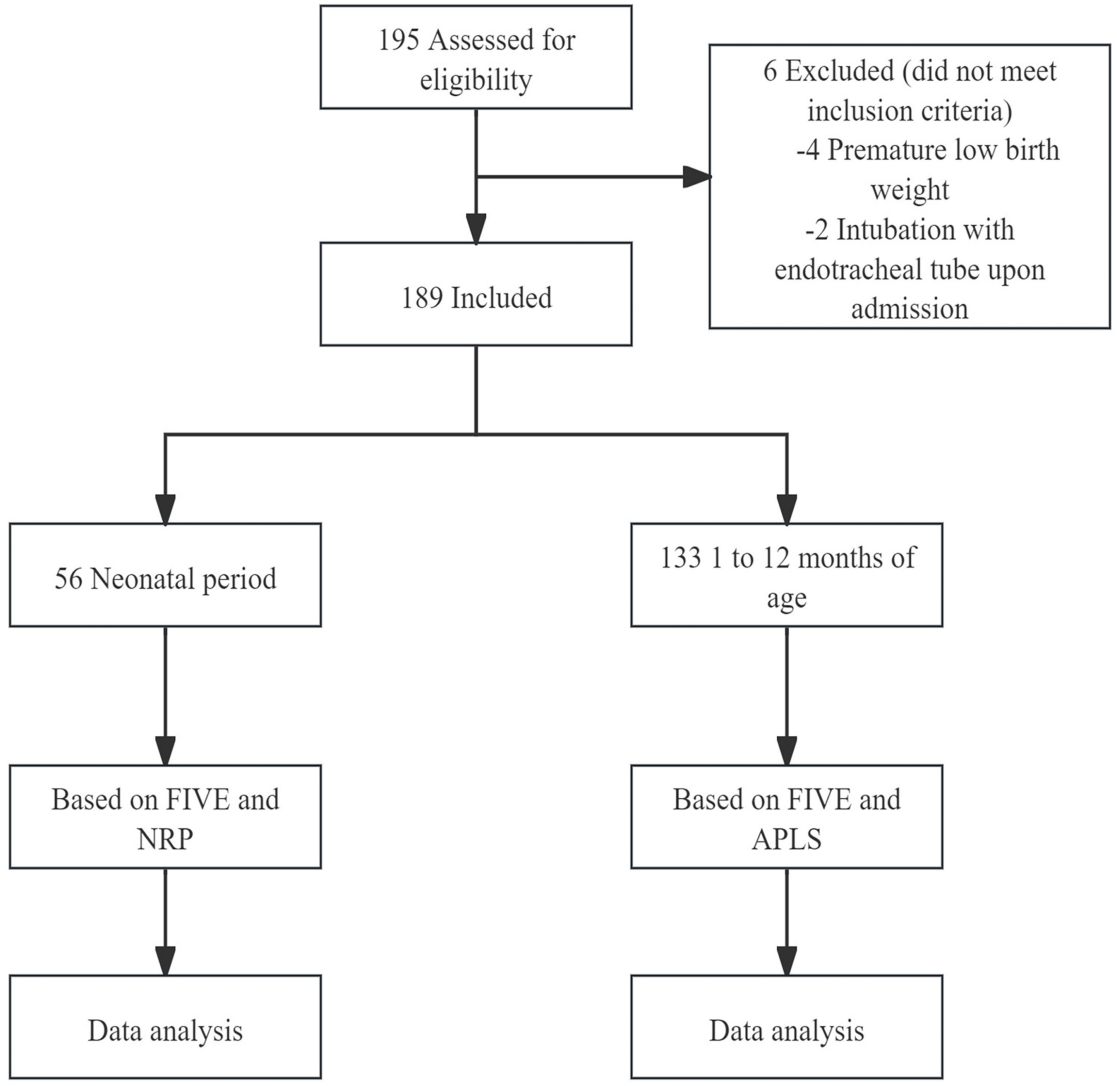
Flow diagram of study participants.

A total of 195 children aged 0–12 months were initially enrolled in this study, of whom 6 were excluded (Figure 2). The final analysis included 189 participants, comprising 56 neonates and 133 infants. In the neonatal subgroup, gestational age ranged from 37^+1^ to 41^+2^ weeks, and birth weight ranged from 2.5 to 3.8 kg. The general characteristics of participants in each age group-including age in months, sex, weight, height, BSA, and tracheal intubation depth-are summarized in Table 1.

**TABLE 1:** General characteristics of infants by age group.

Age (months)	Number of cases	Gender (M/F)	Weight (kg) mean ± SD [min–max]	Height (cm) mean ± SD [min–max]	BSA (m^2^)	FIVE intubation depth (cm)	Formula intubation depth (cm)
Neonatal period	56	31/25	3.4 ± 0.6 [2.5–4.5]	50.5 ± 2.1 [46.2–56.3]	0.22 ± 0.02	9.5 (9.0, 9.5)	9.0 (9.0, 10.0)
1 month of age	38	26/12	4.8 ± 0.7 [3.6–6.5]	55.0 ± 3.2 [50.1–62.5]	0.27 ± 0.03	10.0 (10.0, 10.0)	10.5 (10.0, 10.5)
2 months of age	19	11/8	5.4 ± 0.9 [4.0–7.3]	58.0 ± 2.9 [53.0–66.3]	0.29 ± 0.03	10.0 (10.0, 10.5)	11.0 (10.0, 11.0)
3 months of age	14	5/9	6.1 ± 1.0 [5.2–8.5]	60.4 ± 3.2 [54.3–66.2]	0.32 ± 0.03	10.5 (10.5, 11.0)	11.0 (10.5, 11.0)
4 months of age	7	5/2	7.4 ± 1.5 [5.8–9.5]	62.3 ± 5.7 [55.5–70.8]	0.36 ± 0.05	10.5 (10.5, 11.5)	11.5 (11.0, 12.5)
5 months of age	13	7/6	7.9 ± 1.2 [6.0–9.8]	65.2 ± 2.7 [62.2–71.1]	0.38 ± 0.03	11.0 (11.0, 11.5)	12.0 (11.5, 12.5)
6–12 months of age	42	27/15	9.2 ± 1.3 [6.9–11.9]	70.2 ± 3.0 [64.0–75.5]	0.42 ± 0.04	11.5 (11.5, 12.0)	12.5 (12.0, 13.0)

### 3.1 Comparison of the accuracy of ETT positioning between the formula method and FIVE guidance

Based on intubation depths estimated using the NRP and APLS guidelines, excessively deep intubation occurred in 12.5% of neonates and 30.1% of infants aged 1–12 months (Table 2).

**TABLE 2:** Comparison of the accuracy of ETT positioning between the formula method and FIVE guidance.

Age (months)	Number of cases	Depth conformity [*n* (%)]		χ^2^ value	*P*-value
		Appropriate	Inappropriate		
Neonatal period	56	49 (87.5)	7 (12.5)	5.486	0.019
1–12 months of age	133	93 (69.9)	40 (30.1)	44.755	<0.001

### 3.2 Spearman analysis of related biological factors and intubation depth under FIVE guidance

**FIGURE 3:**
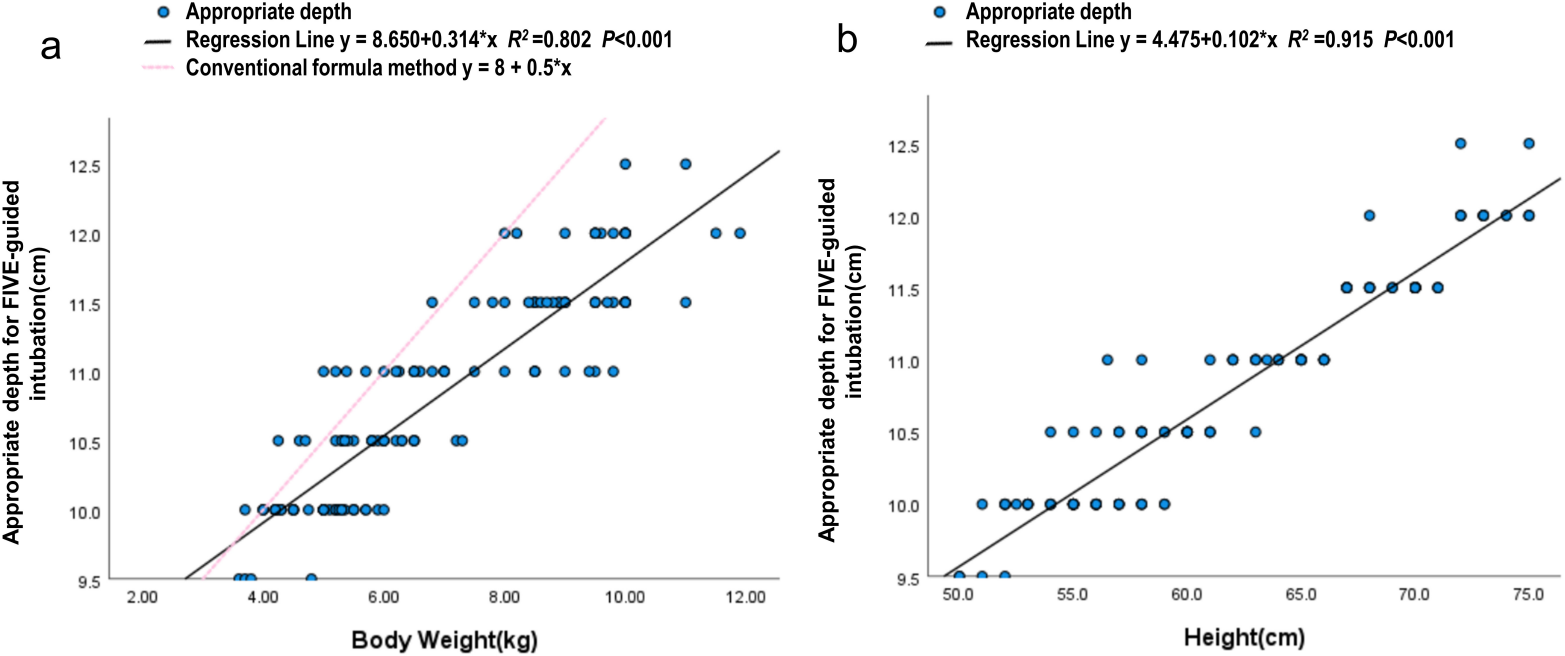
Correlation of weight and height with intubation depth under FIVE guidance in infants aged 1–12 months. **(a)** The regression line shows *y* = 8.650 + 0.314 × x, *R*^2^ = 0.802, *P* < 0.001. **(b)** The regression line shows *y* = 4.475 + 0.102 × x *R*^2^ = 0.915 *P* < 0.001.

Spearman analysis showed that the accuracy of intubation depth under FIVE guidance was significantly correlated with height, age in months, weight, and BSA. In neonatal patients, the correlations were height (*r* = 0.670, *P* < 0.001), weight (*r* = 0.488, *P* < 0.001), and BSA (*r* = 0.536, *P* < 0.001). In patients aged 1–12 months, the correlations were height (*r* = 0.952, *P* < 0.001), weight (*r* = 0.895, *P* < 0.001), BSA (*r* = 0.926, *P* < 0.001), and age in months (*r* = 0.871, *P* < 0.001). Additionally, no correlation was found between gender and intubation depth (Figure 3 and Table 3).

**TABLE 3:** Spearman analysis of gender, age in months, height, weight, and BSA with intubation depth under FIVE guidance.

Biological factors	Neonatal period		1–12 months of age	
	*r*-value	*P*-value	*r*-value	*P*-value
Age in months			0.871*	<0.001
Gender	0.140	0.303	−0.043	0.622
Weight	0.488*	<0.001	0.895*	<0.001
Height	0.670*	<0.001	0.952*	<0.001
BSA	0.536*	<0.001	0.926*	<0.001

*Indicates a highly significant correlation at the *P* < 0.001 level. Weak correlation: r < 0.4; moderate correlation: 0.4 ≤ r < 0.7; strong correlation: r ≥ 0.7.

### 3.3 Prediction using the new formula

Based on the results of the regression analysis, height demonstrated the highest coefficient of determination (*R*^2^) compared with other growth parameters (age, weight, and BSA). Therefore, height was selected as the primary independent variable for predicting optimal endotracheal intubation depth. A height-based linear regression equation was established as follows (Table 4).

**TABLE 4:** Establishment of a new intubation depth prediction formula using linear regression.

Age group (months)	Average intubation depth (cm)	*R* ^2^	Regression analysis (cm)	Predicted intubation depth formula (cm)
Neonatal period	9.45	0.491	3.688 + 0.114 × Height (cm)	4.0 + 0.1 × Height (cm)
	9.45	0.260	8.452 + 0.297 × Weight (kg)	
1–12 months of age	10.81	0.915	4.475 + 0.102 × Height (cm)	4.5 + 0.1 × Height (cm)
	10.81	0.802	8.650 + 0.314 × Weight (kg)	

### 3.4 Bland-Altman agreement analysis between FIVE-guided intubation depth and formula-based calculated intubation depth

**FIGURE 4:**
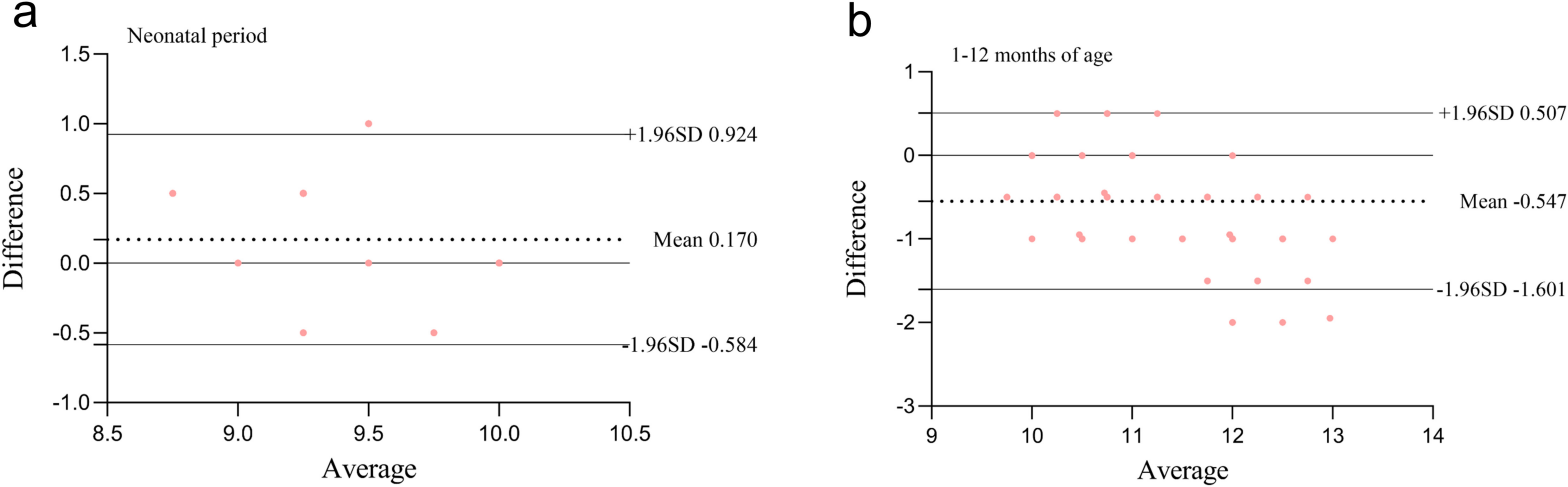
Bland-Altman agreement analysis between FIVE-guided intubation depth and formula-based calculated intubation depth. **(a)** Neonatal period; **(b)** 1–12 months of age.

For neonates, the mean_diff between FIVE-guided intubation depth and the NRP formula-calculated intubation depth was 0.170, with a 95% CI of (0.067, 0.273). The LOA ranged from −0.584 to 0.924, with *P* = 0.002. For patients aged 1–12 months, the mean_diff between FIVE-guided intubation depth and the APLS formula-calculated intubation depth was −0.547, with a 95% CI of (−0.639, −0.455). The LOA ranged from −1.601 to 0.507, with *P* < 0.001. There was no agreement between the two methods, indicating that the APLS formula cannot replace FIVE-guided intubation depth (Figure 4).

## 4 Discussion

In this study, regression analysis identified height as the most significant factor influencing the FIVE-referenced tracheal intubation depth in children aged 0–12 months, followed by BSA and weight. These findings were obtained in stable term neonates and infants intubated in the operating room for elective surgery, and may not generalize to emergency, neonatal intensive care unit (NICU), or critically ill populations. Based on the regression results, a new height-based predictive model was developed to estimate the reference ETT depth for Chinese infants under 1 year of age. The newly developed tracheal intubation depth formulas are as follows: for infants aged 1–12 months, intubation depth (cm) = 4.5 + 0.1 × height (cm); for neonates, intubation depth (cm) = 4.0 + 0.1 × height (cm).

Compared with weight and BSA, height was found to be a more reliable predictor of FIVE-referenced tracheal intubation depth. In the present study, height demonstrated a strong positive correlation with ETT depth in children aged 0–12 months, consistent with previous reports (4, 16, 17). The differences between Lee’s (16) results and our findings may be attributed to differing methodologies and criteria; their formulas were based on positioning the ETT tip at the mid-trachea as determined by CT imaging, whereas our study used FIVE guidance to ensure placement 1 cm above the carina. Currently, there is no universal consensus on the optimal ETT tip position in infants. Neunhoeffer et al. (10) recommended that, on chest radiography, the tip should lie between 0.5 cm above the carina and at least 0.5 cm below the vocal cords. In contrast, Khanna et al. (18) using FIVE to measure tracheal length, advocated positioning the ETT in the middle third of the trachea. In this study, we placed the ETT tip 1 cm above the carina under FIVE guidance (9), the corresponding insertion depth was then rounded down to the nearest 0.5 cm increment according to the tube marking at the incisors. The optimal ETT tip position in neonates/infants is not universally agreed upon and may vary with patient size and head/neck position. We therefore used a prespecified pragmatic target (1 cm above the carina) as the reference position for agreement analyses rather than implying a definitive “ideal” location. Future prospective studies should evaluate alternative target margins (or an acceptable zone above the carina) and determine whether different targets are associated with improved clinical outcomes.

In this study, the APLS weight-based formula demonstrated a high rate of over-insertion (30.1%), indicating that it may systematically overestimate the optimal intubation depth for Chinese infants. This formula was originally developed using data from Western populations and may not adequately account for ethnic and population-specific physiological differences (19). For instance, Chinese infants may have distinct body proportions and airway morphologies compared with their Western counterparts, potentially introducing bias when the APLS formula is applied in this population. Furthermore, because the APLS formula relies solely on weight, its accuracy may be influenced by factors such as nutritional status, fluid retention, or other conditions affecting body mass (20), thereby limiting its ability to accurately reflect tracheal length.

Previous studies have reported that the peak incidence of ETT malposition in infants under 1 year of age can be as high as 35% (21). In addition to the APLS and NRP recommendations, current methods for estimating optimal ETT insertion depth include formula-based approaches derived from age or BSA (5, 10, 16), depth markings on the tube, ultrasound localization (13), chest radiography, and FIVE (22). Compared with the APLS formula, FIVE-guided assessment may help estimate ETT insertion depth and identify potential over-insertion at the time of intubation. In theory, avoiding over-insertion may reduce the likelihood of carinal contact and inadvertent mainstem bronchial intubation. However, the present study evaluated agreement in estimated depth rather than patient outcomes; prospective outcome studies are required to determine whether these approaches translate into fewer adverse events. Over-insertion may cause the tube tip to irritate the carina, increasing the need for sedatives or muscle relaxants, and potentially leading to mucosal injury, granuloma formation, or tracheal scarring (23). Unintended intubation of a mainstem bronchus can result in over-ventilation of one lung, causing emphysema or pneumothorax, while the contralateral lung may collapse (atelectasis) due to under-ventilation, which can severely impair oxygenation and ventilation (24). Given these risks, accurate depth estimation is essential. The formula developed in this study, derived from data on Chinese infants under 1 year old, may provide more accurate predictions of ETT depth in this population than existing formulas. Regardless of the calculation method used, clinicians must also consider factors such as the type of surgery (25, 26), patient body position and head/neck alignment (27). After intubation, ETT placement should be verified by a combination of chest auscultation, observation of chest wall movement, visualization of tube misting, end-tidal carbon dioxide monitoring, and pulse oximetry. As height is the primary predictor in our model, accurate and standardized height measurement is essential when applying the formula. In our study, height was measured using a calibrated infantometer with a two-person technique and repeat measurements to minimize variability; however, residual measurement error remains possible. Because a 1–2 cm error in height could translate into an approximately 1–2 mm difference in the predicted ETT depth, we recommend using the formula in conjunction with routine clinical confirmation of tube position, and future external validation studies should adopt a standardized height-measurement protocol (device, positioning, and repeat measurements) to ensure reproducibility across centers.

Peng et al. (8) demonstrated that the Tochen formula (ETT depth [cm] = weight [kg] + 6) (28) estimates intubation depth more accurately than the NRP-recommended chart in Asian neonates, providing a useful reference for clinical practice in Chinese infants. However, Chung et al. (29) reported an over-insertion rate of 64.7% when using the NRP-recommended Tochen formula, likely related to the high proportion of preterm (62%) and low birth weight (54%) infants in their cohort. In the present study, even after excluding preterm and low birth weight neonates, 12.5% still experienced over-insertion. Although methods based on weight, gestational age, and nasal septum to ear tragus length (8) are widely used clinically, none can accurately predict the optimal ETT length for neonates (30). Despite Bland-Altman analysis revealing a statistically significant difference (*P* = 0.002) in intubation depth between the NRP and FIVE methods for neonates, the mean difference of merely 0.170 cm was considered clinically negligible, indicating good clinical agreement between the two approaches. Recent guidelines recommend video laryngoscopy as the preferred method for neonatal intubation (1). Nevertheless, the neonatal trachea is short-approximately 4 cm-and tracheal length correlates closely with gestational age and birth weight (31), so even small depth adjustments (e.g., 0.5 cm) (32) can significantly increase the risk of malposition. The formula ETT depth (cm) = 4.0 + 0.1 × height (cm) may serve as a reference, but in clinical practice, depth should be individualized based on gestational age, height, weight, and other patient-specific factors to reduce the likelihood of over-insertion and should be verified using standard clinical checks. Ultrasound’s inability to precisely determine the ETT’s intracheal position (13) is further compounded by its accuracy being influenced by various factors. These include subjective image interpretation, a lack of standardized procedures, and the operator’s experience (33, 34). While chest radiography can confirm ETT tip position, it is time-consuming, exposes patients to ionizing radiation, and is impractical for routine anesthesia. In contrast, FIVE allows direct visualization of the tube tip relative to the carina and was used as the reference method for depth determination in this study (22). Importantly, because clinical outcomes were not assessed, these potential benefits remain theoretical and should be evaluated in prospective outcome studies.

This study has several limitations. First, it was conducted at a single center; therefore, multicenter studies with larger, more diverse cohorts are needed to validate our findings and assess the performance of the new formula across different patient populations. In addition, the predictive equation was developed and evaluated within the same cohort, and no independent external validation cohort was available. This may lead to optimistic performance estimates due to overfitting; thus, the formula should be considered preliminary and requires external validation. Second, the applicability of our formula to preterm and low-birth-weight neonates has not been validated; therefore, it should not be directly applied to these populations without dedicated validation. Third, the applicability of the new formula was not evaluated in infants with airway malformations or conditions such as congenital tracheal stenosis or laryngomalacia, whose airway anatomy may differ substantially from normal and thus affect predictive accuracy. Fourth, all intubations were performed in the operating room under general anesthesia; therefore, our findings may not be directly generalizable to non-anesthesia settings [e.g., pediatric intensive care unit (PICU)/NICU or emergency intubations], where patient physiology, airway conditions, and clinical practice may differ. Finally, although the new formula demonstrated higher accuracy than existing guidelines, it remains an estimation tool and may not provide precise predictions for every individual case.

## 5 Conclusion

For stable infants undergoing elective surgery in the authors’ region, the APLS formula was associated with a higher proportion of depths classified as over-insertion when compared with FIVE-guided depth. We derived a height-based formula for Chinese infants aged 1–12 months: intubation depth (cm) = 4.5 + 0.1 × height (cm), which showed closer agreement with the FIVE-referenced depth than existing guideline formulas in this cohort. Because this equation was developed and assessed within the same single-center cohort, it should be considered preliminary and requires independent external validation before broader use, particularly in non-elective or critically patients.

## Data Availability

The original contributions presented in this study are included in this article/supplementary material, further inquiries can be directed to the corresponding authors.
